# Utilization of Self-Consolidated Green Material for Sustainable Development: An Environment Friendly Waste Materials Application for Circular Economy

**DOI:** 10.3390/polym13172985

**Published:** 2021-09-03

**Authors:** Syyed Adnan Raheel Shah, Hassam Ahmad, Hatem Alhazmi, Muhammad Kashif Anwar, Fahad Iqbal

**Affiliations:** 1 Department of Civil Engineering, Pakistan Institute of Engineering & Technology, Multan 66000, Pakistan; hassamahmad04@gmail.com (H.A.); kashifanwar723@gmail.com (M.K.A.); 2 National Center for Environmental Technology (NCET), Life Science and Environment Research Institute (LSERI), King Abdulaziz City for Science and Technology (KACST), Riyadh 11442, Saudi Arabia; halhazmi@kacst.edu.sa; 3 Department of Mechanical and Structural Engineering and Materials Science, University of Stavanger, NO-4036 Stavanger, Norway; fahadmeyo@gmail.com

**Keywords:** sustainability, circular economy, waste materials, mechanical properties, Self-Compacting Concrete

## Abstract

Self-Compacting Concrete (SCC) is a unique kind of concrete that tends to consolidate in terms of its weight. In this study, the prime target is to investigate the durability properties of SCC developed using eco-friendly economical waste binding materials as partial replacement to costly cement. This circular economy concept will not only help in the development of green concrete but will also help to improve the climatic condition by reducing the use and production of cement. An economical design methodology has been applied to produce environmentally friendly construction material. This research focuses on the application of Alum Sludge (AS) and Brick Dust (BD) in Self-Compacting Concrete (SCC). Both materials are waste materials containing binding properties. Performance of SCC developed using these two materials was tested considering mechanical properties of concrete using the destructive testing technique. Results showed that BD and AS can be utilized for up to 12% and 9% of replacement of cement, respectively, to achieve equal or higher compressive, tensile, and flexural strength. The application of BD and AS has demonstrated a subsequent improvement of SCC’s mechanical properties, i.e., compressive, tensile, and flexural strength. This study will help the production of composite green materials with the help of eco-friendly and economical waste materials for sustainable infrastructure development.

## 1. Introduction

Self-Compacting Concrete (SCC) has a different behavior to that of normal concrete used around the globe in the construction industry. The effective self-consolidating capability of developed concrete without any external mechanical vibrations makes it much more powerful and useful in the construction industry. Its use of conveniently inexpensive and environment-friendly products is a great achievement. It is utilized in places where the vibration mechanism is not easy to install, and concrete composite material needs vibration to remove air bubbles to retain strength. It is also of great advantage to integrate and substitute many different waste products into the materials being used in the construction industry by increasing their partial replacement. Bricks are the major and most important type of utilized building material used around the globe and are a main part of this construction industry. With such large-scale production, there becomes a generated waste outcome in the form of brick dust or surkhi. In the conventional approach, prepared bricks are usually essentially masonry units made of simple inorganic or non-metallic materials that are fully sundried or burned in kilns to develop strength. Burnt bricks have a much stronger compressive strength property compared to sundried ones. Brick Dust (BD) is the waste dust material produced during burnt brick creation. It is used primarily as an effective substitute for concrete cement replacement. Thousands of tons of brick waste powder are generated every year around the globe, and it remains untreated and mishandled. BD mainly functions like sand as a filler, but it effectively imparts higher strength and invented hydraulic properties to self-compact concrete [1,2]. BD is usually an organic but pozzolanic product formed by the powdering bricks or the burnt clay balls produced by the kiln burning. The introduction of BD into concrete as a substitute for cement completely reduces the high cost of using cement and decreases cement used in the construction industry, leading to the development of an environmentally friendly but viable growth in the construction industry. It induces concrete resistance to alkalis and harmful salt solutions.

Alum Sludge (AS) is essentially a by-product produced worldwide that is derived from water treatment units and plants that use aluminum salts as primary coagulants. It has chemical composition of Al_2_O_3_, which also a part of cement; therefore, it is an established fact that it also possesses binding properties and can be used as binder in construction process. Alum Sludge is the world’s most widely developed residual water treatment. Alum Sludge is essentially made up of colloidal alum hydroxides, usually used amorphous material. The current studies concentrate on the major impact of Alum Sludge powder as an active partial replacement of Portland cement and study the related mechanical properties of self-compacting concrete with high performance [3,4]. Sieve analysis was done to elaborate the size of alum powder with a percentage of 90 percent fineness to check its feasibility relevant to cement. The current study used the M20 concrete mix with a *w*/*c* of 0.40. For the inclusion of Alum Sludge and Brick Dust in SCC, different proportions of substitution have been exercised. Selected percentages of the overall cement content used were 5, 10, 15, 20, 25, and 30%. Samples are cast to evaluate the compressive strengths and substitution effects on concrete mixes’ fresh properties. At 7, 14, and 21 days of age, specimens are cured and screened for both hardened and fresh properties. 

In the trend to use industrial waste and by-products as an effective useful substitution of ingredients utilized in the construction industry, rapid growth is observed. This approach gives an effective and environmentally sustainable superiority to erection development around the globe [5,6,7].

## 2. Background

Waste is becoming a serious public health hazard around the world. On the other hand, recognizing and utilizing it as viable raw material sourcing for the construction industry would improve resource efficiency because implementing such an approach could result in the development of a Circular Economy (CE) system that closes the material loops. As a result, natural resource removal is minimized, the carbon footprint is reduced, and waste is eliminated [8,9,10,11,12]. In the first phase, the raw constituents are made after manufacturers are supplied with the appropriate raw ingredients and residual by-products of different sources (i.e., GGBFS, FA, and SF) to replace conventional cement (OPC) and reduce waste dumping. This is then addressed by construction techniques and the building’s service life. When the building reaches the end of its lifespan, destruction will occur, and the waste created will be reused for the same or another activity. The structure should be renovated and maintained as necessary to enhance its lifespan.

**Figure 1:**
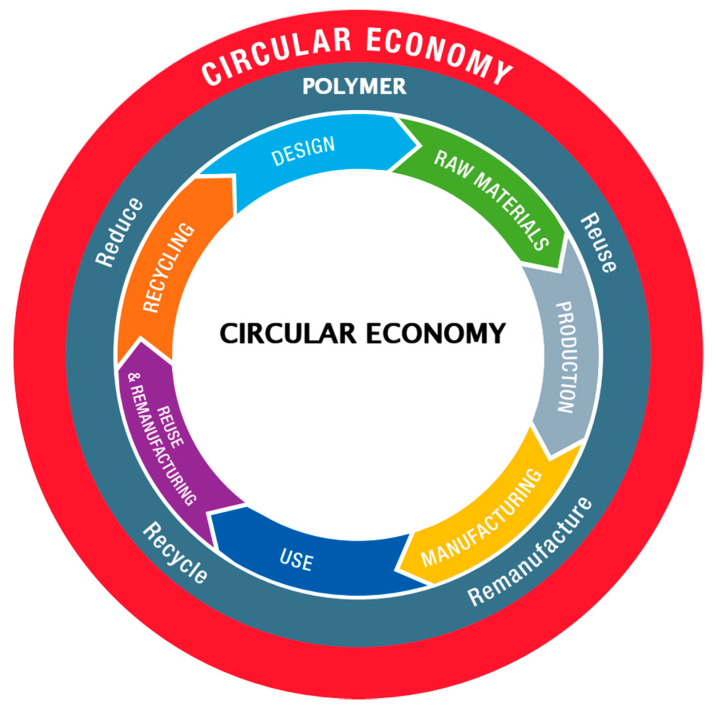
Circular Economy Cycle for Sustainable Development [15].

Green material has often been considered an important aspect in enhancing the aspects of sustainability: ecological, economical, and social [13]. This will take place due to the circularity property of green concrete processes as it will help to reduce carbon footprints as well as utilizing waste materials to save dumping capacity and costs [10]. As a result, adopting an eco-friendly concrete that uses waste as one of its constituents to partially substitute conventional cement could play a key role in developing a facility that improves structural knowledge while also making it eco-sustainable and cost-effective. Additionally, dumping such waste materials and by-products in landfill areas is a significant environmental challenge since they contain a substantial amount of leachable harmful components that can affect the ecosystem by contaminating the water, soil, or air [14]. The circular economy follows the basic principle of utilizing waste from one source as a product for another source. The circular economy formation for the sustainable cyclic process is elaborated on in Figure 1.

## 3. Materials and Methods

### 3.1. Major Ingredients

#### 3.1.1. Brick Dust

BD is created when bricks and brick kilns are loaded or unloaded where they are burned. Since ancient times, pozzolanic materials such as brick dust and other powder ceramics have been used in concrete. Bricks are generally made of large Clay materials consisting of about 20–30% Alumina, about 50–60% Silica, and various other oxides and carbonates. 

Clay is truly responsible for Surkhi’s most successful pozzolanic behavior. Tile used to produce bricks does not have a substantial pozzolanic property in scope, but it develops a visible pozzolanic quality when it is fired with other agents such as lime during the brick manufacturing process [16,17,18,19].

#### 3.1.2. Chemical Reaction in Concrete Brick Dust

Brick Dust or Surkhi behaves as pozzolana and responds as an outcome with the lime in an active water presence. If carbon dioxide reacts effectively with calcium hydroxide in the process, calcium carbonate (CaCO_3_) and water are formed as a result. The most important chemical reactions are as follows:

Water + Portland Cement(Type-1) → Calcium Silicate Hydrate(1)

CO_2_ + Ca(OH)_2_ → H_2_O + CaCO_3_ (2)

When it reacts with the lime, the extra amount of useful hydraulic cement is formed. The response is as follows:

Ca(OH)_2_ + Pozzolana + water → C-H-S (Glue) (3)

The former reaction is a very rapid reaction in nature that efficiently provides a measurable early strength enhancement for the concrete, whereas the second reaction of pozzolana with completely released lime in the presence of water is usually a sluggish reaction in nature that affects the strengths of concrete in the early age to some degree [20]. However, with the passage of healing time, the Surkhi (brick dust) effectively increases the intensity of SCC by producing an extra quantity of C-H-S that increases the binder properties to a greater extent.

#### 3.1.3. Alum Sludge (AS)

In the current study, AS is introduced in concrete to be utilized as a sustainable ingredient to replace cement up to 30 percent. It is mixed with ordinary Portland cement to boost strength and elevate the base mechanical assets of concrete for the growth and production of self-compacting concrete with high performance [21,22,23,24].

**Figure 2:**
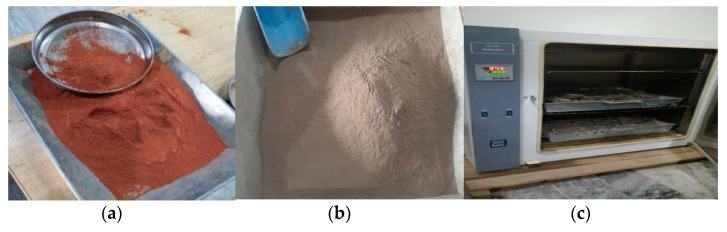
(**a**) Brick Dust (**b**) Powdered Alum (**c**) Oven for Drying.

The Alum Sludge was collected primarily from the treatment plant during the water treatment process and was then fully oven-dried at approximately 105 °C, as shown in Figure 2.

A Los Angeles abrasion test device was then used to finely grind the oven-dried alum sludge to a powder. The Alum was ground until it achieved the required 905 fineness. Then it was sieved correctly using a sieve ring of 45 μm to achieve optimal fineness. Alum sludge’s basic chemical compositions have been studied [25]. The Alum Sludge’s extensive chemical composition and all its physical properties are summarized as shown in Table 1.

#### 3.1.4. Super-Plasticizer

The primary ingredient of a Self-compacting concrete (SCC) is “Superplasticizer” because it is also an essential part of SCC concrete. Superplasticizers are utilized to form workable concrete in circumstances where the placing of concrete is unapproachable or in circumstances where fast employment is vital [26]. By incorporating admixtures as a crucial portion of the mix in the shape substitution, they soak up water for the manufacture of high-strength concrete, thereby increasing the water volume as well as eventually impeding the workability as water is utilized to accomplish the hydration procedure.

These superplasticizers are condensates of sulfonated melamine formaldehyde or formaldehyde condensates of sulfonated naphthalene [27]. The plasticizer used in the research is the “Ultra Chemicals Super Plasticizer” and it is the favored “Sulfonated Naphthalene Formaldehyde Condensate” due to its prominent dispersing action on concrete. The sulfonic acid in it helps to disperse the particles of cement by adsorbing them and producing a negative charge on them that makes them mutually repulsive [28]. Conversely, these plasticizers’ costs are very high because their industrial procedure and ingredients are very costly [29].

#### 3.1.5. Cement

The Standard Portland Cement of the Grade 53 has been used. The cement was truly clean, new, and free of lumps and unwanted impurities.

#### 3.1.6. Fine Aggregates

The fine aggregate of the Lawrencepur type was used to develop the sample. It was passed through sieve #4 carefully. The effective fineness module of the used fine aggregate has been defined by ASTM C 136-93 [29,30,31,32].

#### 3.1.7. Coarse Aggregates

Coarse aggregates are a significant part of concrete and are used in the current District Abbottabad “Margalla” crush studies. Coarse aggregates are analyzed following the ASTM C 136 standard through sieve testing.

### 3.2. Concrete Preparation and Testing

#### 3.2.1. Development of Concrete

Self-compacting concrete is a useful mix prepared in ordinary traditional concrete by induction of a superplasticizer. SCC has been created out of social necessity, like many innovative products. To test and create the most effective and beneficial combination, various ratios are chosen. The amount of plasticizer varies from 2–3% depending upon the intensity of workability and slump. Just applying the plasticizer after adding water to the mix improves the attributes of decent workability, and the simple positioning for SCC is equipped close to ordinary concrete. It performs the J-circle, V-funnel, fresh mix, and slump tests. To prepare the SCC, a standard mechanical mixer is used. Because of its high mobility, it is much easier to handle and place than standard concrete [4,29]. In the mixer machine, fresh concrete is prepared along with the replacement of ordinary cement, which is done during the mixing process. SF, GGBFS, and MP are mixed with 150 mm by 150 mm by 150 mm, 100 mm by 100 mm by 500 mm prisms, and 150 mm by 300 mm cylinders are cast. After de-moulding, the specimens are cured for 7, 14, 21, and 28 days. After healing, cube samples are dried for 20 min in heated ovens at approximately 300–450 °C. The proportion of mixing is shown in Table 2.

Various experiments were carried out to analyze the mechanical foundation as well as the fresh properties of Alum Sludge and Brick Dust modified SCC concrete. Self-compacting concrete mixes of varying AS and BD percentages are prepared with 0, 3, 6, 9, 12, and 15% substitution of ordinary Portland cement. The blend used was the 1:1.5:3 mixing ratio of the M20 concrete and a 0.40 *w*/*c*. The following is the mix design modified by Alum Sludge (AS) and Brick Dust (BD) for the prepared SCC samples.

#### 3.2.2. Properties of Fresh Concrete

**Figure 3:**
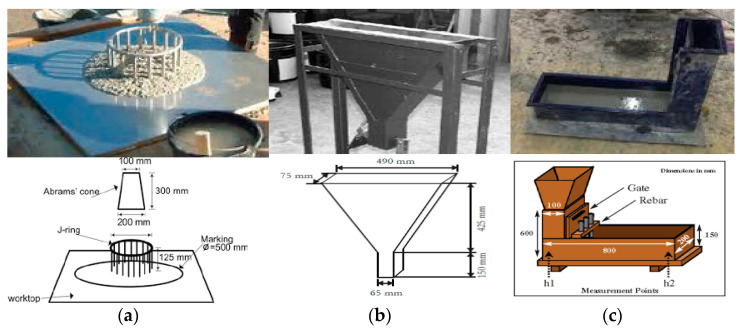
Standard (**a**) J-Ring, (**b**) V-Funnel (**c**) L-Box test apparatus.

Standard SCC fresh property tests were performed, including the standard V-funnel tests, L-Box, and J-ring tests, to explore the fresh properties of SCC concrete [29,33,34,35]. The equipment utilized for testing the above-listed tests is shown in Figure 3.

***J-Ring Test:*** The basic generic J-Ring test was used for the thorough assessment of concrete flow capability for the detailed evaluation of the key fresh assets of updated SCC. The standard J-Ring system available in the laboratory of the institute was used efficiently to perform the test according to the BS requirements shown in Figure 3a.

***V-Funnel Test:*** The generic V-funnel testing tool was employed to perform this experiment efficiently as shown in Figure 3b. The test was conducted in the laboratory and a stopwatch was used to monitor the stream timings. The fundamental and primary aim of the V-funnel test is to allow us to fully evaluate and analyze SCC concrete’s key flow pattern through the reinforcement bars in a structure.

***L-Box Test:*** One of the most important and useful SCC tests is the “L-box test,” which was applied effectively to review and calculate the passing competence of the full filling and concrete. This test is accurate and applicable to the required concrete character of high flow capability, as shown in Figure 3c.

The detailed evaluation of the fresh properties of the prepared SCC mixes with Alum Sludge and Brick Dust are shown in Table 3.

The results indicated in Table 3 demonstrate that SCC with added Alum Sludge and Brick Dust possessed normal values for all the fresh properties tests, but upon the increment of added percentages, more than 15% of both the materials induced decreased workability nature. A good range of values is obtained for 9% and 12% replacements. Previously, a series of materials had similar ranges of the utilization of different materials [36,37].

#### 3.2.3. Properties of Hardened Concrete

**Figure 4:**
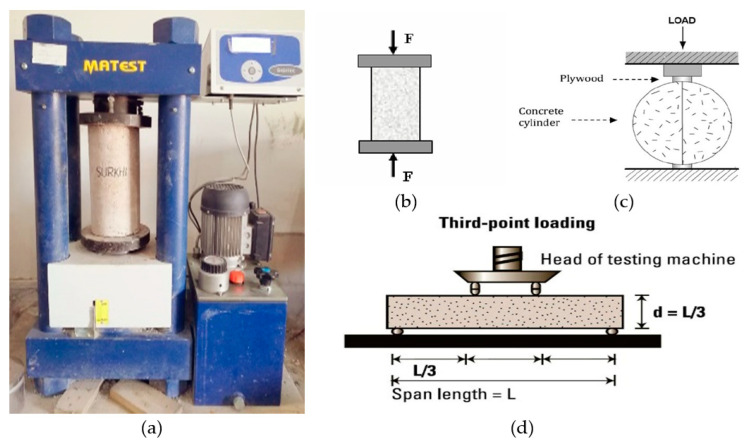
(**a**) Testing machine, (**b**) compressive strength test mechanism, (**c**) split tensile test mechanism, and (**d**) flexural strength test mechanism.

The primary characteristics that are considered for judging the quality, strength, and degradation of concrete are its mechanical properties (i.e., compressive strength, split tensile strength, and flexural strengths) [29]. They also depend upon the mix ratio, volume of aggregate, and the physical characteristics of materials [38]. Hardened characteristics play a vital role in the study of evolved concrete behavior; there are typically three main tests involved (as shown in Figure 4) in the study of concrete behavior: (i) compression test; (ii) indirect tensile test; (iii) flexural strength test.

Strength testing of concrete materials is identified with substantial burden strength testing. Two kinds of specimens were delivered to quantify substantial strength: block (600 × 600 × 600) and chamber (600 × 1200). Likewise, the Indirect Tension test, otherwise called the Brazilian and Japanese test, is done to gauge the upward impact of the compressive burden on the vertically stacked chamber to test its rigidity. A compressed wood strip is mounted at the mark of utilization to lessen the higher effect of tension on the substantial surface. The flexural examination is done on a shaft substantial example. There ought to be, for the most part, some substantial obstruction before breaking begins, and the heap is moved to reinforcing. The get-together displayed in Figure 4 is a three-point bending system that can take breaks at any piece of the beam. By estimating substantial quality through these three measures, the concrete strength and activities under changing conduct can be effortlessly assessed.

## 4. Results

### 4.1. Compression Strength

**Figure 5:**
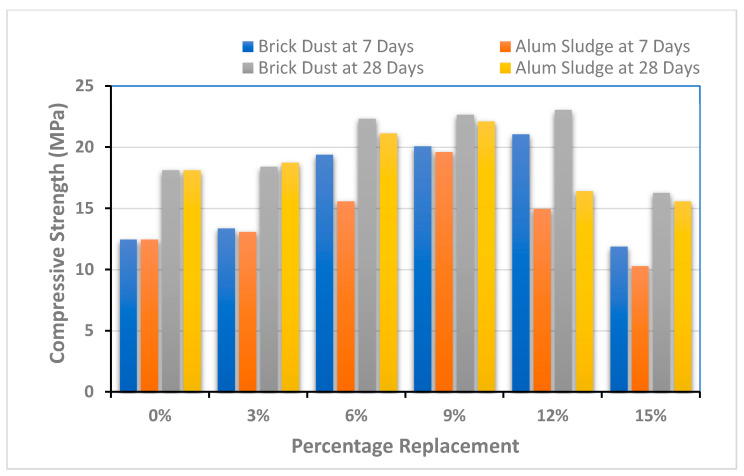
Compressive strength performance of composite material.

Samples were checked for compressive strength determination, as shown in Figure 5. Two specimens were developed and tested for each ratio (five ratios) and substance (two materials). The action of brick dust (BD) has been found better compared to alum sludge (AS) with the percentage increase. Additionally, the literature shows that the strength of concrete has varied by utilizing these additives. Figure 5 below displays the values of compressive strength in modified SCC for AS and BD. The testing results of compressive strength revealed that incorporation of BD up to 12% by weight of cement improved and enhanced the mechanical properties of SSC. SCC has shown a strength of 12.46 MPa for the control sample at 7 days strength and 18.14 MPa for the control sample at 28 days strength. However, the addition of the optimum level of BD at 12% and AS at 9% showed improvement, and strength increased up to 21.05 MPa and 19.63 MPa for 7 days and 23.04 MPa and 22.13 MPa for 28 days curing, respectively. Likewise, the addition of BD and AS showed enhanced compressive strength properties up to 12% and 9% by weight of cement. The reason is that both materials induced the binding properties of self-compacted concrete, which leads to improved strength properties. The maximum compressive strength for 28 days of curing has been found at 23.04 MPa, which is more than 21% as compared to the control specimen by incorporating 12% of BD with the weight of the cement. Similarly, the maximum compressive strength has been achieved by more than 18% with the incorporation of AS at about 9% by the total weight of the cement at 28 days curing. Therefore, a recommended material as a partial replacement of cement can be Brick Dust. 

### 4.2. Split Tensile Strength

**Figure 6:**
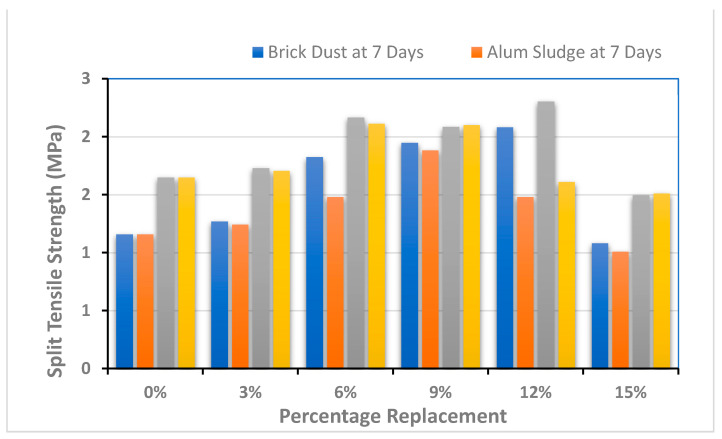
Split tensile strength performance of composite material.

One of the most important features of tensile strength is that concrete is highly vulnerable to cracking. The standard cylinder specimen is laid between the compression machine plates, and then the behavior of the specimen is noted along the perpendicular diameter. Three specimens were developed and tested for each ratio (five ratios) and substance (two materials), as shown in Figure 6.

The testing results of split tensile strength are shown in Figure 6. The bar charts of Figure 6 demonstrate that the split tensile strength is increased initially with up to 12% and 9% replacement of BD and AS, a substitute to cement. The value of the tensile strength leads to a decrease with any more addition of these materials. SCC has shown the tensile strength of 1.16 MPa for the control sample at 7 days strength and 1.65 MPa for the control sample at 28 days strength. However, the addition of the optimum level of BD at 12% and AS at 9% showed improvement, and strength increased up to 2.08 MPa and 1.88 MPa for 7 days and 2.30 MPa and 2.10 MPa for 28 days curing, respectively. Likewise, the addition of BD and AS showed enhanced tensile strength properties up to 12% and 9% by weight of the cement. The maximum tensile strength was 2.30 MPa, which was found to be increased 28% more than the control sample by incorporating 12% of brick dust, whereas the maximum tensile strength with the addition of 9% of alum sludge as a replacement to cement was more than 21% when compared to the control sample. 

The action of BD has been found to be better when compared to its other competitor alum sludge with the percentage increase. The virtual analysis illustrates that concrete strength can be tested, as shown in Figure 6, and there is very little difference in the highest strength values.

### 4.3. Flexural Strength

**Figure 7:**
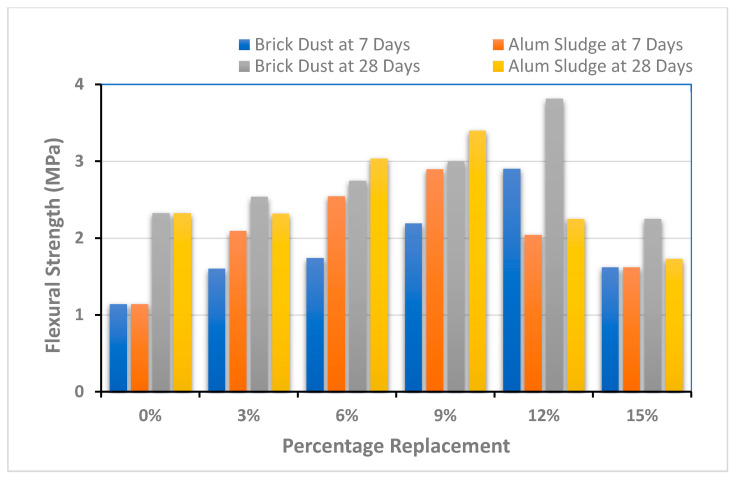
Flexural strength performance of composite material.

Flexural strength is one measure of concrete’s tensile strength. To test flexural strength, a concrete beam’s resistance to loading is assessed. Three specimens were developed and tested for each ratio (five ratios) and substance (two materials). These beams were subjected to a charging system of three stages. It is important to see the comparable flexural strength in Figure 7 for comparative analysis.

Graphical representation of flexural strength results is shown in Figure 7 and demonstrate that the flexural strength is increased initially with up to 12% and 9% replacement of BD and AS a substitute to cement. The value of flexural strength leads to a decrease with any more addition of these materials. SCC has shown a flexural strength of 1.14 MPa for the control sample at 7 days strength and 2.33 MPa for the control sample at 28 days strength. However, the addition of optimum levels of BD at 12% and AS at 9% showed improvement, and strength increased up to 2.90 MPa and 2.89 MPa for 7 days and 3.81 MPa and 3.40 MPa for 28 days curing, respectively. Likewise, the addition of BD and AS showed enhanced tensile strength properties up to 12% and 9% by weight the of cement. The maximum flexural strength was 3.81 MPa, which was found to have increased 64% more than the control sample by incorporating 12% of brick dust, whereas the maximum tensile strength with the addition of 9% of alum sludge as a replacement to cement was more than 46% when compared to the control sample. Hence, a recommended material as a partial replacement of cement can be Brick Dust.

## 5. Discussion

The testing results of mechanical properties such as compressive strength, split tensile strength, and flexural strength are shown in Figure 5, Figure 6 and Figure 7. Two specimens for each ratio (five ratios) and substance (two materials) were prepared to evaluate each mechanical property. Then, the average or mean value is calculated for better results analysis. From the above graphs, it has been found that the incorporation of BD shows significant results as compared to AS. The results of both brick dust and alum sludge-based mix of SCC have shown enhanced mechanical properties as compared to control specimens up to a certain limit.

The testing results of compressive strength shown in Figure 5 revealed that incorporation of BD up to 12% by weight of the cement improved and enhanced the mechanical properties of SCC. Likewise, the addition of AS shows enhanced strength properties up to 9% by weight of the cement. The reason is that both materials induced the binding properties of self-compacted concrete, which leads to improved strength properties. SCC has shown the strength of 12.46 MPa for the control sample at 7 days strength and 18.14 MPa for the control sample at 28 days strength. However, the addition of the optimum level of BD at 12% and AS at 9% showed improvement, and the strength increased up to 21.05 MPa and 19.63 MPa for 7 days and 23.04 MPa and 22.13 MPa for 28 days curing, respectively. It was concluded that a certain percentage of these waste materials up to the optimum level of 12% (for BD) and 9% (for AS) may lead to a successful contribution in the compressive strength.

The testing results of split tensile strength are shown in Figure 6. The bar charts of Figure 6 demonstrate that the split tensile strength is increased initially with up to 12% and 9% replacement of BD and AS a substitute to cement. SCC has shown the tensile strength of 1.16 MPa for the control sample at 7 days strength and 1.65 MPa for the control sample at 28 days strength. However, the addition of the optimum level of BD at 12% and AS at 9% showed improvement, and the strength increased up to 2.08 MPa and 1.88 MPa for 7 days and 2.30 MPa and 2.10 MPa for 28 days curing, respectively. It was concluded that a certain percentage of these waste materials up to the optimum level of 12% (for BD) and 9% (for AS) may lead to a successful contribution in the indirect tensile strength.

The findings of flexural strength are presented in the bar charts of Figure 7, which show increasing flexural strengths by the addition of BD at all percentage levels except 15%. SCC has shown flexural strength of 1.14 MPa for the control sample at 7 days strength and 2.33 MPa for the control sample at 28 days strength. However, the addition of the optimum level of BD at 12% and AS at 9% showed improvement, and the strength increased up to 2.90 MPa and 2.89 MPa for 7 days and 3.81 MPa and 3.40 MPa for 28 days curing, respectively. With these testing results, BD and AS both can be considered as potential partial replacements of cement for SCC development and utilization.

The results concluded that with the increase of AS replacement percentage, both the compressive and split tensile strengths increased, but only up to a certain limit of 9% addition. After that, with increased ratios, the effective compressive strengths decreased due to the increased AS content and low cement content that caused lesser hydration reactions and failed to form the binding jell to hold aggregates in strength [39,40]. Brick Dust increased the effective strengths with up to 15% incorporation into the concrete, while after that, it showed a slight decrease. 

Application of the developed SSC using BD and AS can be helpful as an economical solution and effective utilization of waste materials for construction. This combination of materials will serve as a low-cost solution to the construction industry and follows the principles of sustainable development goals.

## 6. Conclusions

In the current research conducted upon the Alum Sludge and Brick Dust that were incorporated in SCC by an effective partial replacement of the ordinary Portland cement, we came can develop the following comprehensive conclusions based on the results obtained after deep evaluation by testing.

- The maximum workability trend was noted to be at 15% replacement of cement with Brick Dust and Alum Sludge, and after that, it decreased.
- The compressive strength pattern for the addition of BD in SCC tends to elevate by increasing the content until 12%. Beyond this point, the replacement increase affected the compressive strengths.
- The split tensile strengths of SCC samples increased by increasing the content of BD up to the addition of 12%, while after this, it effectively tends to decrease the associated enhancements.
- Alum Sludge (AS) is a useful by-product that comes out as a waste from the water treatment plants. The workability behavior of AS modified SCC remained normal to the generally observed trend, and it increases slightly with increased percentages.
- The concrete with 9% AS shows a significantly improved strength gain across all curing ages.
- With the utilization of both waste materials, concrete development can take a step forward to achieving a sustainable yet low-cost and durable construction trend in our construction industry.

## 7. Recommendation and Future Directions

The proposed modification of binding material with AS and BD can not only help in the development of concrete, but can also be used in mortar formation, and at some stage, with the help of chemical scientists, a modified low-cost cement can be developed, which can not only help in low cost-economical material composite formation but will also reduce the construction cost. In the future, this combination can also help in the development of different types of composite materials for large infrastructure projects like dams. This will help in the reduction of construction costs.

## Figures and Tables

**Table 1:** External properties and aluminum sludge chemical composition.

Chemical Analysis, (%)										Physical Tests	
SiO_2_	Al_2_O_3_	Fe_2_O_3_	CaO	MgO	SO_3_	Na_2_O	K_2_O	P_2_O_5_	OI	Specific gravity	Moisture content (%)
42.38	35.03	4.94	0.13	0.29	0.14	0.10	1.87	0.26	11.86	2.34	0.85

**Table 2:** Mixing and development rates for building.

Mix ID	Cement kg/m^3^	AS kg/m^3^	BD kg/m^3^	CA kg/m^3^	FA kg/m^3^	Water kg/m^3^	SP kg/m^3^
CSSC0%	410	-	-	1278	648	164	4.7
AS5%	389.5	20.5	-	1278	648	164	4.7
AS10%	369	41	-	1278	648	164	4.7
AS15%	348.5	61.5	-	1278	648	164	4.7
AS20%	328	82	-	1278	648	164	4.7
AS25%	307.5	102.5	-	1278	648	164	4.7
AS30%	287	123	-	1278	648	164	4.7
BD5%	389.5	-	20.5	1278	648	164	4.7
BD10%	369	-	41	1278	648	164	4.7
BD15%	348.5	-	61.5	1278	648	164	4.7
BD20%	328	-	82	1278	648	164	4.7
BD25%	307.5	-	102.5	1278	648	164	4.7
BD30%	287	-	123	1278	648	164	4.7

**Table 3:** Fresh properties of the prepared SCC mixes with Alum Sludge and Brick Dust.

Material	Fresh Properties			
Binder Replacement	Slump Test	V-Funnel Test	J-Ring Test	L-Box
(%)	(mm)	(s)	(mm)	(H2/H1)
CS 0%	689	9.4	36	0.90
AS 3%	742	10.1	27	0.90
AS 6%	672	9.86	32	0.88
AS 9%	710	9.61	29	0.87
AS 12%	710	9.52	35	0.85
AS 15%	690	10.1	33	0.82
BD 3%	735	9.98	37	0.90
BD 6%	765	10.1	31	0.85
BD 9%	773	9.98	32	0.84
BD 12%	767	9.74	35	0.81
BD 15%	742	9.88	28	0.72
**Standard**	**650–800**	**6–12**	**≤50**	**0.8–1.0**

Note: CS, Control sample; AS, Alum Sludge; BD, Brick Dust.

## Data Availability

Data will be made available on suitable demand.

## References

[1] Bourgeois J., Walsh M., Gagnon G. (2004). Treatment of drinking water residuals: Comparing sedimentation and dissolved air flotation performance with optimal cation ratios. Water Res..

[2] Fytianos K., Voudrias E., Raikos N. (1998). Modelling of phosphorus removal from aqueous and wastewater samples using ferric iron. Environ. Pollut..

[3] Guan X.-H., Chen G.-H., Shang C. (2005). Re-use of water treatment works sludge to enhance particulate pollutant removal from sewage. Water Res..

[4] Haque M., Kayali O. (1998). Properties of high-strength concrete using a fine fly ash. Cem. Concr. Res..

[5] Sotero-Santos R.B., Rocha O., Povinelli J. (2007). Toxicity of ferric chloride sludge to aquatic organisms. Chemosphere.

[6] Tay J.-H. (1987). Sludge ash as filler for Portland cement concrete. J. Environ. Eng..

[7] Tay J.-H., Show K.-Y. (1992). Utilization of municipal wastewater sludge as building and construction materials. Resour. Conserv. Recycl..

[8] Marie I., Quiasrawi H. (2012). Closed-loop recycling of recycled concrete aggregates. J. Clean. Prod..

[9] Colorado H.A., Velásquez E.I.G., Monteiro S.N. (2020). Sustainability of additive manufacturing: The circular economy of materials and environmental perspectives. J. Mater. Res. Technol..

[10] Knoeri C., Sanyé-Mengual E., Althaus H.-J. (2013). Comparative LCA of recycled and conventional concrete for structural applications. Int. J. Life Cycle Assess..

[11] Leising E., Quist J., Bocken N. (2018). Circular Economy in the building sector: Three cases and a collaboration tool. J. Clean. Prod..

[12] Al-Hamrani A., Kucukvar M., Alnahhal W., Mahdi E., Onat N.C. (2021). Green Concrete for a Circular Economy: A Review on Sustainability, Durability, and Structural Properties. Materials.

[13] Suhendro B. (2014). Toward Green Concrete for Better Sustainable Environment. Procedia Eng..

[14] Rashad A.M. (2015). An investigation of high-volume fly ash concrete blended with slag subjected to elevated temperatures. J. Clean. Prod..

[15] PCE (2021). Polymer Circular Economy. https://www.protoolplast.com/wp-content/uploads/2018/11/polymer-circular-economy.png.

[16] Hassan A.A., Lachemi M., Hossain K.M. (2012). Effect of metakaolin and silica fume on the durability of self-consolidating concrete. Cem. Concr. Compos..

[17] Melo K.A., Carneiro A.M. (2010). Effect of Metakaolin’s finesses and content in self-consolidating concrete. Constr. Build. Mater..

[18] Su N., Hsu K.-C., Chai H.-W. (2001). A simple mix design method for self-compacting concrete. Cem. Concr. Res..

[19] Krstulović P., Kamenić N., Popović K. (1994). A new approach in evaluation of filler effect in cement I. Effect on strength and workability of mortar and concrete. Cem. Concr. Res..

[20] Yılmaz B., Ediz N. (2008). The use of raw and calcined diatomite in cement production. Cem. Concr. Compos..

[21] Ozawa K. (1994). Evaluation of self-compactability of fresh concrete using the funnel test. Doboku Gakkai Ronbunshu.

[22] Neville A.M., Brooks J.J. (1987). Concrete Technology.

[23] Kodur V., Sultan M. (2003). Effect of temperature on thermal properties of high-strength concrete. J. Mater. Civ. Eng..

[24] Saad M., Abo-El-Enein S., Hanna G., Kotkata M. (1996). Effect of temperature on physical and mechanical properties of concrete containing silica fume. Cem. Concr. Res..

[25] Phan L.T., Lawson J.R., Davis F.L. (2001). Effects of elevated temperature exposure on heating characteristics, spalling, and residual properties of high performance concrete. Mater. Struct..

[26] SCCEP Group (2005). The European Guidelines for Self-Compacting Concrete: Specification, Production and Use, International Bureau for Precast Concrete (BIBM). http://www.efca.info/efca-publications/concrete-construction/.

[27] Okamura H., Ouchi M. (2003). Self-compacting concrete. J. Adv. Concr. Technol..

[28] Persson B. (2001). A comparison between mechanical properties of self-compacting concrete and the corresponding properties of normal concrete. Cem. Concr. Res..

[29] Wibowo N.A. (2019). Designing of Flow Mortar Design Mix for Self-Compacting Concrete (SCC) with FWC = 0.4. J. Civ. Eng. Forum.

[30] Alyamaç K.E., Ince R. (2009). A preliminary concrete mix design for SCC with marble powders. Constr. Build. Mater..

[31] Topcu I.B., Bilir T., Uygunoğlu T. (2009). Effect of waste marble dust content as filler on properties of self-compacting concrete. Constr. Build. Mater..

[32] Corinaldesi V., Moriconi G. (2003). The influence of mineral additions on the rheology of self-compacting concrete. Spec. Publ..

[33] Zardi M., Rahmawati C., Azman T.K. (2016). Pengaruh Persentase Penambahan Sika Viscocrete-10 Terhadap Kuat Tekan Beton. J. Teknik Sipil Unaya.

[34] Celik M.Y., Sabah E. (2008). Geological and technical characterisation of Iscehisar (Afyon-Turkey) marble deposits and the impact of marble waste on environmental pollution. J. Environ. Manag..

[35] Güneyisi E., Gesoǧlu M., Özturan T., Mermerdaş K. (2012). Microstructural properties and pozzolanic activity of calcined kaolins as supplementary cementing materials. Can. J. Civ. Eng..

[36] Duan P., Shui Z., Chen W., Shen C. (2013). Effects of metakaolin, silica fume and slag on pore structure, interfacial transition zone and compressive strength of concrete. Constr. Build. Mater..

[37] Siddique R. (2007). Waste Materials and By-Products in Concrete.

[38] Widodo S. (2004). Optimalisasi Kuat Tekan Self-Compacting Concrete dengan Cara Trial-Mix Komposisi Agregat dan Filler pada Campuran Adukan Beton. J. Penelit. Saintek.

[39] Larbi J.A. (1991). The Cement Paste-Aggregate Interfacial Zone in Concrete. https://repository.tudelft.nl/islandora/object/uuid:db49e8b6-db0b-4724-b00f-2ea187e8c244?collection=research.

[40] Khan S.U., Nuruddin M.F., Ayub T., Shafiq N. (2014). Effects of Different Mineral Admixtures on the Properties of Fresh Concrete. Sci. World J..

